# Supplementary material for: Identification of Freezing-Responsive microRNAs and Their Targets in Chinese Jujube by Small RNA and Degradome Sequencing
Source: Int J Mol Sci. 2026 Sep 4;27(17):7912. doi: 10.3390/ijms27177912 (PMC13566890; doi:10.3390/ijms27177912)
Supplement: Supplementary file 1 [file ijms-27-07912-s001.zip › Supplementary Material S2/novel_miR_178.pdf]

Provisional ID	:	novel_miR_178
Score total	:	0.4
Score for star read(s)	:	-1.3
Score for read counts	:	0
Score for mfe	:	0.1
Score for randfold	:	1.6
Score for cons. seed	:	
Total read count	:	57
Mature read count	:	57
Loop read count	:	0
Star read count	:	0

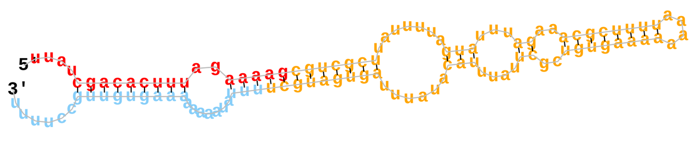

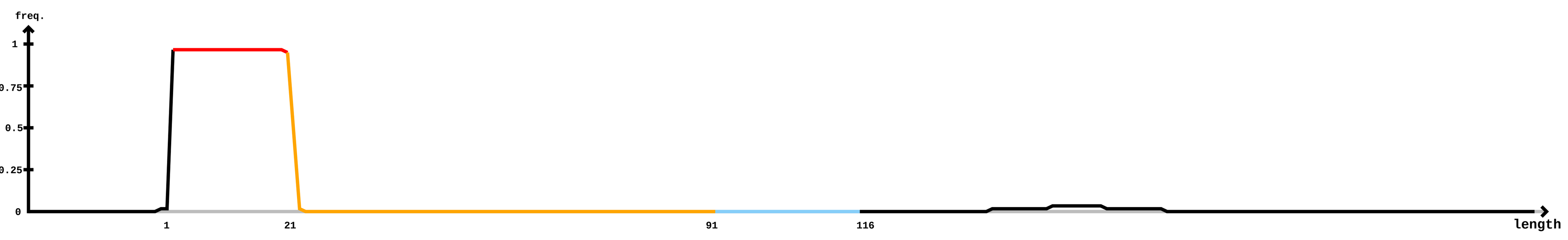

Mature   Star

```
5'- gaaaaguauguuauccuaauauuuaucgacacuuuagaaaagcgucgcuuauuuuaguauuuagaaacgcuuuuaaaaaaaagugucgcuuauuuacauauuuagugaugcuuuuauaaaaaaaaguguugccuuuuauuuuuauuuuauuuuuccuuccaauuugaaaguugcuuuuaguuuucucaaauuaugacuguuuaauaguuaaauucaugcaaaauaauuaauagacaacaacguucaaaua  -3'   exp
    (((((((...((....((((.....((((((((..(((((((((((.......(((...((..((((((((....))))))))..))....)))......)))))))))))).......))))))))).....))))..))...))))))))......(((((((.((((((.((((((....((((((.(((((((...)))))))))))))..)).....)))))).))....)))).))))))).  reads  mm  sample
    ....................auuuaucgacacuuuagaaGagcg..............................................................................................................................................................................................................  1  1  LT-3
    ......................uuaucgacacuuuagaaaU.................................................................................................................................................................................................................  1  1  LT-3
    ......................uuaucgacacuuuagaaaag................................................................................................................................................................................................................  27  0  LCK-2
    ......................uuaucgacacuuuNgaaaag................................................................................................................................................................................................................  1  1  LCK-1
    ......................uuaucgacacuuuagaaaag................................................................................................................................................................................................................  26  0  LCK-1
    ......................uuaucgCcacuuuagaaaag................................................................................................................................................................................................................  1  1  LCK-2
    ..............................................................................................................................................................ccGauuugaaaguugcuuu.........................................................................  1  1  LT-3
    ........................................................................................................................................................................aguugcuuGuaguuuucuc...............................................................  1  1  LCK-1
```
